# Impact of a deletion of the full-length and short isoform of p75NTR on cholinergic innervation and the population of postmitotic doublecortin positive cells in the dentate gyrus

**DOI:** 10.3389/fnana.2015.00063

**Published:** 2015-05-27

**Authors:** Robert Poser, Martin Dokter, Viola von Bohlen und Halbach, Stefan M. Berger, Ruben Busch, Marian Baldus, Klaus Unsicker, Oliver von Bohlen und Halbach

**Affiliations:** ^1^Institute of Anatomy and Cell Biology, Universitätsmedizin GreifswaldGreifswald, Germany; ^2^Department of Molecular Biology, Central Institute of Mental Health and Medical Faculty Mannheim, Heidelberg UniversityMannheim, Germany; ^3^Department of Molecular Embryology, Institute of Anatomy and Cell Biology, University of FreiburgFreiburg, Germany

**Keywords:** neurotrophin, p75NTR, adult neurogenesis, dendritic spine, cholinergic system

## Abstract

Analyses of mice carrying a deletion of the pan-neurotrophin receptor p75NTR have allowed identifying p75NTR as an important structural regulator of the hippocampus. Most of the previous analyses were done using p75NTR^*ExIII*^ knockout mice which still express the short isoform of p75NTR. To scrutinize the role of p75NTR in the hippocampus, we analyzed adult and aged p75NTR^*ExIV*^ knockout mice, in which both, the short and the full-length isoform are deleted. Deletion of these isoforms induced morphological alterations in the adult dentate gyrus (DG), leading to an increase in the thickness of the molecular and granular layer. Based on these observations, we next determined the morphological substrates that might contribute to this phenotype. The cholinergic innervation of the molecular and granular layer of the DG was found to be significantly increased in the knockout mice. Furthermore, adult neurogenesis in the DG was found to be significantly altered with increased numbers of doublecortin (DCX) positive cells and reduced numbers of apoptotic cells in p75NTR^*ExIV*^ knockout mice. However, cell proliferation as measured by phosphohiston H3 (PH3) positive cell numbers was not affected. These morphological alterations (number of DCX-positive cells and increased cholinergic fiber densities) as well as reduced cell death in the DG are likely to contribute to the observed thickening of the granular layer in p75NTR^*ExIV*^ knockout mice. In addition, Sholl-analysis of DCX-positive neurons revealed a higher dendritic complexity and could thus be a possible morphological correlate for the increased thickness of the molecular layer in p75NTR deficient animals. Our data clearly demonstrate that deletion of both, the short and the full-length isoform of p75NTR affects DG morphology, due to alterations of the cholinergic system and an imbalance between neurogenesis and programmed cell death within the subgranular zone.

## Introduction

The neurotrophins are a family of growth factors that includes brain-derived neurotrophic factor (BDNF), nerve growth factor (NGF), neurotrophin-3 (NT-3) and neurotrophin-4/5 (NT-4/5). Neurotrophins exert many of their specific actions through tyrosine kinase (trk) receptors that bind the neurotrophins with high affinity and, in addition, all neurotrophins can signal through a low-affinity receptor, known as p75NTR (see for review: Chao, 2003). Moreover, p75NTR seems to represent a high-affinity receptor for proNGF (see for review: Underwood and Coulson, 2008). The p75NTR is a transmembrane protein composed of an extracellular domain containing several cysteine-rich motifs, a transmembrane domain, and a non-catalytic intra-cellular domain (Underwood and Coulson, 2008). Two isoforms of the p75NTR exist: a short (s-p75NTR) and a full-length isoform. The full-length isoform is capable of binding neurotrophins, whereas the short isoform lacks the neurotrophin binding site. Although the functions of s-p75NTR are largely unknown, some studies suggest that it is a functional receptor *in vivo* (Fujii and Kunugi, 2009).

In order to analyze the functions and roles of p75NTR in more detail, p75NTR knockout mice have been generated. One knockout mouse line has been created by deleting Exon 3, which encodes parts of the extracellular domain (Lee et al., 1992). The homozygote knockout mice (which will be referred to as p75NTR^*ExIII*^) are viable and fertile; however they display several defects in the nervous system, including, among others, deficits in the peripheral sensory nervous system (Lee et al., 1992) and loss of sensory neurons in the dorsal root ganglia (Murray et al., 1999). As a consequence of alternative splicing, p75NTR^*ExIII*^ knockout mice are hypomorph because they still express s-p75NTR (Nykjaer et al., 2005). Another p75 knockout line was generated by von Schack et al. (2001) carrying a deletion of Exon IV (referred to as p75NTR^*ExIV*^). Deletion of Exon IV results in a loss of both, the full-length and the short isoform of p75NTR (von Schack et al., 2001). About 40% of the homozygous p75NTR^*ExIV*^ knockout mice die during the late fetal or early postnatal period, and surviving mice display impaired motility during the first postnatal weeks because of hind limb ataxia (von Schack et al., 2001).

In the adult brain, p75NTR is expressed, *inter alia*, within the hippocampus (Barrett et al., 2005), including the dentate gyrus (DG). Several studies have assessed the putative impact of p75NTR on hippocampal morphology, neurogenesis and hippocampus-related behavior (Wright et al., 2004; Catts et al., 2008; Bernabeu and Longo, 2010; Colditz et al., 2010). However, all these studies were done on p75NTR^*ExIII*^ deficient mice and provided contradictionary results (summarized e.g., in Dokter et al., 2015). The conflicting reports on hippocampal structure and behavior of p75NTR^*ExIII*^ deficient mice may be attributed to the application of diverse methods used for analysis as well as to the genetic background of the p75NTR^*ExIII*^ deficient mice and their controls. In addition to the hippocampal phenotype, cholinergic neuron numbers and innervation density were shown to be altered in p75NTR^*ExIII*^ knockout mice (Van der Zee et al., 1996; Yeo et al., 1997; Naumann et al., 2002; Neseliler et al., 2011). Interestingly, in p75NTR^*ExIV*^ knockout mice the increase in cholinergic neurons was reported to be higher, as compared to p75NTR^*ExIII*^ knockout mice (Naumann et al., 2002). Moreover, it has been shown that hippocampal CA1 pyramidal neurons cultured from p75NTR^*ExIV*^ mice display a higher increase in spine density than neurons from p75NTR^*ExIII*^ mice (Zagrebelsky et al., 2005). Despite these different morphological changes in area CA1, both, p75NTR^*ExIII*^ and p75NTR^*ExIV*^ knockout mice show similar impairments in hippocampal long-term depression, whereas long-term potentiation (LTP) in both mutants is unaffected (Rösch et al., 2005).

However, it can be speculated that deletion of p75NTR has also an impact upon the DG, since proNGF can signal via p75NTR and since proNGF seems to be capable of inhibiting adult neurogenesis in the DG (Guo et al., 2013). Concerning adult neurogenesis in the DG, it should also be noted that intraventricular injections of 192-IgG saporin in rat severely reduced hippocampal cholinergic innervation and also reduced the number of doublecortin immunoreactive neurons in the DG (Fréchette et al., 2009). Moreover, there are data hinting that acetylcholine may promote neurogenesis (Mohapel et al., 2005). Thus, deletion of p75NTR may affect both, cholinergic innervation of the hippocampus as well as adult neurogenesis. Since the impact of a knockout of p75NTR^*ExIV*^ upon the DG has not been examined in detail yet, we were therefore interested to analyze whether p75NTR^*ExIV*^ knockout mice display an altered morphology of the DG. In detail we were interested to see whether (1) the neurotransmitter supply in the hippocampus is affected, whether (2) spine densities of DG granule cells are altered, and (3) whether changes in adult hippocampal neurogenesis can be observed in p75NTR^*ExIV*^ mice.

## Material and Methods

### Animals

p75NTR^*ExIV*^ knockout mice and control littermates were breed from heterozygous mice. These heterozygous mice were offsprings of the strain generated by von Schack and coworkers, 2001 (von Schack et al., 2001). Homozygous knockout mice (p75NTR^*ExIV−/−*^) were analyzed in comparison to littermate controls (p75NTR^*ExIV+/+*^) that were both obtained by crossing heterozygous p75NTR^*ExIV*^ mice.

For the subsequent analysis, mice with an age of 4–6 months (“adult”) as well as with an age between 20 and 23 months (“aged”) were used. All animal experiments were performed in accordance with German animal rights regulations and with permission of the Landesamt für Landwirtschaft, Lebensmittelsicherheit und Fischerei (LALLF) Mecklenburg-Vorpommern, Germany.

### Histology

Animals were killed with an overdose of ether and transcardially perfused with phosphate-buffered saline (PBS: 2.0 g NaH_2_PO_4_, 10.73 g Na_2_HPO_4_ and 9.0 g NaCl in 1,000 ml distilled water, pH 7.2) followed by perfusion with 4% paraformaldehyde (PFA; dissolved in PBS, pH 7.2). Brains were removed and immersed in the same fixative for at least 5 days.

### Assessment of Layer Width of the DG

The width of the granule cell layer and the molecular layer of the DG was measured on a series of 4′,6-diamidino-2-phenylindole (DAPI; Molecular Probes, Leiden, Netherlands; 1:10,000) stained 30 μm thick coronal serial sections, sampled at a random start position [around Bregma −1.34 mm, determined using a mouse brain atlas (Paxinos and Franklin, 2001)]. Per section the width of the upper blade of the granule layer and the molecular layer and of the DG was measured at three different points. A mean of 10 sections was analyzed per animal (thus 30 measurements per region and animal). The width was calculated as the average across all measured sections using NeuroLucida (MBF Biosciences, USA).

### HPLC

From homozygous knockout mice and respective littermate controls brains were removed and frozen in isopentane/dry ice. From 120 μm thick frozen brain sections obtained by using a cryostat (Leica, Nußloch, Germany), tissue samples from hippocampus were collected using specific punching needles. For quantification of monoaminergic neurotransmitters and their metabolites, tissue samples were homogenized in an extraction solution (0.1 M perchloric acid, 1 mM EDTA) using the tissue homogenizer Mixer Mill (Qiagen, Hilden, Germany) and yielded homogenates centrifuged at 15,000 g for 10 min at 4°C. 10 μl of supernatant was applied on a HPLC system with electrochemical detection, consisting of an Antec LC-100 isocratic pump (Shimadzu, Duisburg, Germany), a Spark Triathlon autosampler (Spark Holland, Emmen, The Netherlands), a C18-OptiAqua reverse phase column (150 × 2.1 mm; 3 μm particle size) (Techlab, Braunschweig, Germany) and a Decade II electrochemical detector (Antec Leyden, Zoeterwoude, The Netherlands). The mobile phase contained 50 mM sodium citrate, 2.1 mM octyl sodium sulfate, 0.1 mM EDTA, 10 mM NaCl, and 23% methanol at pH 4.0. The temperature applied on the system for optimal peak separation was 37°C. Using this protocol, concentrations of dopamine (DA), noradrenaline (NA), serotonin (5-HT) 3, 4-dihydroxyphenylacetic acid (DOPAC), 5-hydroxyindoleacetic acid (5-HIAA) and homovanillic acid (HVA) can be quantified simultaneously by determining the area under each peak using the software Clarity (Data Apex, Prague, Czech Republic) and referencing it the an appropriate standard curve. All tissue concentrations were calculated by normalizing the quantified neurotransmitter amounts to the respective weight of the tissue sample.

### Determination of Cholinergic Fiber Densities

30 μm thick coronal sections were made using a vibratome and collected in 20% ethanol. On the next day, slices were incubated in sodium citrate buffer (pH 6.0) for 20 min using a microwave (700 W) for antigen retrieval. Thereafter, sections were incubated for 72 h with polyclonal goat anti choline-acetyltransferase (ChAT) antiserum (1:200; Millipore, USA) in the presence of 0.1% Triton-X100. After rinsing the sections were transferred to biotinylated anti-goat IgG (1:200; Vector, Burlingame, USA) for 2 h at room temperature. After washing sections were incubated in Cy3-conjugated streptavidin (1:2,000; Jackson Immunoresearch, USA) for 2 h at room temperature. Sections were counterstained with DAPI (1:10,000), washed, and coverslipped in fluorescent mounting medium (DAKO, USA).

Fiber densities were quantified using an Axioplan 2 imaging microscope (Zeiss, Germany). The randomly chosen microscope field of interest (ROI: 50 × 50 μm) either located within the upper leaf of the subgranular layer (SGZ) or the molecular layer of the DG was captured by an AxioCam video camera (Zeiss, Germany), connected to a personal computer. A grid consisting of single pixels spaced 2.5 × 2.5 μm apart (in the x- by y-plane) was overlaid on the image and fibers intercepting grid points were counted. Relative fiber densities were expressed as Q = G_i_/G_o_. G_i_ is the number of fibers intercepting the grid points and G_o_ is the total number of grid points within the region of interest (von Bohlen und Halbach, 2013). At least six different regions of interest (starting at around Bregma −1.46 mm) were analyzed per zone and animal at different rostro-caudal positions of the DG, spaced 120 μm (in the z-axis) apart.

### Activated Caspase-3 Staining

For determination of apoptotic cell death, we used antibodies directed against activated caspase-3 (Freund et al., 2014). Coronal sections of 30 μm thickness were mounted and air-dried over night. On the next day, slices were incubated in sodium citrate buffer (pH 6.0) for 20 min using a microwave (700 W) for antigen retrieval. After this, sections were washed and incubated in blocking solution (0.1 M PBS, 0.3% Triton X-100, 2% serum) for 1 h at RT. Thereafter, sections were incubated in a solution containing rabbit antibodies (1:250) directed against (active) caspase 3 (AB3623, Millipore, Germany) over night at 4°C. After washing, sections were incubated in a solution containing Cy3-conjugated goat anti-rabbit IgG (Vector Labs, USA; 1:400, for 1 h). Sections were counterstained with DAPI (1:10,000), washed and coverslipped in fluorescent mounting medium.

### Adult Neurogenesis

The time-course of adult hippocampal neurogenesis can be subdivided into different stages, during which different markers are expressed (von Bohlen und Halbach, 2011). To examine cell proliferation within the DG, phosphohistone H3 was used as a specific marker; NeuroD was used to label mitotic active neuronal cells, and doublecortin (DCX) was used to label newly formed neurons. Coronal sections of 30 μm thickness (or 60 μm in case for Sholl-analysis) from the entire hippocampus were cut with a vibratome (Leica VT1000, Germany). The following antibodies and substances were used: rabbit α-phosphohistone H3 (Santa Cruz, Germany), goat α-NeuroD (1:100; Santa Cruz, Germany), goat α-doublecortin (DCX; 1:200; Santa Cruz, Germany), biotinylated horse α-goat, (1:200; Vector, Germany), biotinylated goat α-rabbit (1:200; Vector, Germany); streptavidin-Cy3 (1:1,000; Jackson Immunoresearch, USA).

For phosphohistone H3 staining, sections were mounted and air-dried over night. On the next day, sections were washed and then incubated in sodium citrate buffer (pH 6.0) for 20 min using a microwave oven (700 W) for antigen retrieval. After this, sections were washed in a solution containing 0.1 M PBS, 0.3% Triton X-100, 3% serum) for 1 h at RT. For NeuroD staining, sections were mounted and air-dried. Sections were then washed and then incubated in sodium citrate buffer (pH 6.0) for 20 min using a microwave (700 W) for antigen retrieval. After this, sections were incubated in blocking solution (0.1 M PBS, 0.1% Triton X-100, 1% serum) for 1 h at RT. For DCX immunohistochemistry, we used free-floating sections. Sections were incubated for 1 h in a blocking solution containing 0.3% Triton X-100 and 3% bovine serum albumin (BSA) in PBS.

Thereafter, sections were incubated in a solution (0.1 Triton X-100 and 3% serum in PBS) containing antibodies directed either against phosphohistone H3, NeuroD or DCX over night at 4°C. Visualization was done using a biotinylated secondary Cy3-conjugated streptavidin antibody (Jackson Immunoresearch, USA). Sections were counterstained with DAPI (1:10,000) and washed (the DCX stained sections were mounted and air-dried over night) and then coverslipped in fluorescent mounting medium.

### Counting of Labeled Cells

To estimate the number of the labeled cells, cell counts were made using the serial sections, as described previously (Dokter et al., 2015). In brief: countings were performed according to the Abercrombie’s correction formula (starting around Bregma −1.06 mm), since this method renders biases within the range of the optical disector by taking into account that the particles counted are small compared with the section thickness (von Bartheld, 2002). No guard zones were used, since the use of guard zones can bias even optical disector counting (Baryshnikova et al., 2006). The Linderstrom-Lang/Abercrombie (LLA) equation for estimating numerical neuronal densities is:

N = n * t(t + H)orN/n = f = t/ (t + H)

N is an estimate of the number of objects in the defined region, n is the counted number of objects, t is the mean thickness of the virtual section, H is the mean height of the objects, and f is the conversion factor for converting n to N.

In a first step, n was quantified using an Axioplan 2 imaging microscope (Zeiss, Germany) fitted for fluorescence. In a second step, H, the height of the cells in the z-axis, was estimated using a computer-driven motorized stage (Merzhäuser, Germany) connected to the Axioplan 2 imaging microscope (Zeiss, Germany) under the control of StereoInvestigator (MBF Biosciences, USA).

### Sholl-Analysis

For identification and reconstruction of DCX-stained neurons, z-stacks (step-width: 1 μm) were generated. From these z-stacks DCX-positive neurons within the granular layer of the DG were reconstructed using NeuroLucida (MBF Biosciences, USA) and the reconstructions were analyzed with the help of NeuroExplorer (MBF Biosciences, USA) using the module “Sholl analysis” for the analysis of the three-dimensional vector-based Neurolucida data sets. Sholl analysis was performed using 6 p75NTR^*ExIV+/+*^ and 5 p75NTR^*ExIV−/−*^ animals. Per animal, a mean of 10 individual neurons were reconstructed. For visualization of the z-stacks, images were loaded in ImageJ (NIH, USA) and processed using the z-project plug-in (parameter: “Max Intensity”).

#### Analysis of Dendritic Spines

Brains were impregnated according to the Golgi-Cox procedure [using Rapid GolgiStain reagents (FD NeuroTechnologies, USA)] and cut at 120 μm. Analysis of dendritic spines was conducted in a blinded procedure. Only one segment per individual dendritic branch and neuron was chosen for the analysis. Spines were analyzed in the dorsal, but not ventral, leaf of the DG because spine densities are different in these locations and no subdivision into different types of spines [filopodia, stubby, thin, and mushroom, which reflect temporal snapshots of a dynamic phenomenon (Parnass et al., 2000)] was made as outlined previously in detail (von Bohlen und Halbach et al., 2006). Analyses were conducted on Golgi-impregnated sections that were uniformly dark throughout the section. Only dendrites that displayed no breaks in their staining (Leuner et al., 2003) and that were not obscured by other neurons or artifacts (Liu et al., 2001) were evaluated. Three-dimensional reconstruction and evaluation were performed using NeuroLucida and a 100x oil immersions objective, as described previously (Waltereit et al., 2012). Dendrites from granule cells of the DG were used for analysis. At least 30 dendrites per region and animal (n) were reconstructed with approximately 3000 individual spines per animal. The n values for the statistical analysis were based on animal numbers and not on numbers of analyzed elements.

#### Statistics

Statistical analyses were performed using Prism 6.0 for Windows (GraphPad Software Inc., USA). For statistical evaluation of two groups unpaired two-sided *t*-tests were used.

For the analysis of more than two groups one-way ANOVA followed by a Tukey’s multiple comparisons test was used. In case of the HPLC analysis, a two-way ANOVA followed by a Bonferroni post-test was used. Significance levels for all tests were set at *p* ≤ 0.05. Data in figures were expressed as mean ± SEM; significant changes in the figures were indicated by an asterisk.

## Results

### Thickness of the Molecular and Granular Layer of the DG

**Figure 1:**
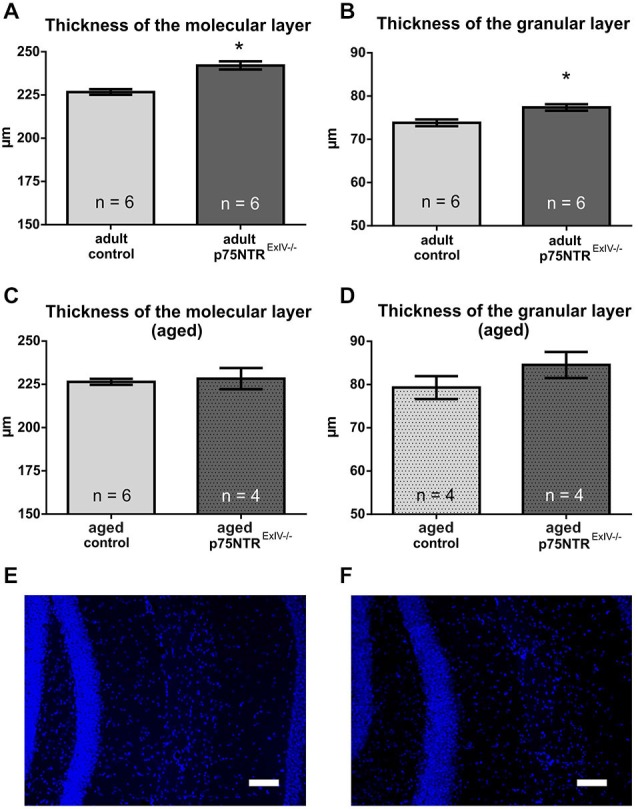
**The morphology of the dentate gyrus (DG) is altered in p75NTR^ExIV−/−^** mice. Compared to age-matched controls, the mean thickness of both, the molecular **(A)** and the granular **(B)** layer of the DG is increased in adult p75NTR^*ExIV−/−*^ mice as. In contrast, aged p75NTR^*ExIV−/−*^ mice show a slight, but insignificant increase in the mean thickness of the molecular **(C)** or granular layer **(D)** of the DG. A representative image of the thickness of granular and molecular layer of the upper leaf of the DG of an control animal is shown in** (E)** and a representative image of the thickness of the granular layer and molecular layer of the upper leaf of the DG of a p75NTR^*ExIV−/−*^ mouse is in** (F)**. Scalebars: 100 μm.

We have previously shown that functional deletion of the long isoform of the p75 receptor (p75NTR^*ExIII*^ knockout) causes a significant increase of the DG resulting from an increased width of the granular, but not the molecular layer in adults (Dokter et al., 2015). Here, we show that deletion of both, the long and the short isoform of the p75 receptor leads to an increase in thickness of the molecular (6.8%; *p* = 0.0004) as well as the granular layer (4.8%; *p* = 0.008) in adult p75NTR^*ExIV*^ knockout mice (Figure 1). Thus, deletion of both, the long and the short isoform of the p75 receptors aggravates the phenotype concerning DG morphology. However, no apparent differences were observed in aged animals, (Figures 1C,D), neither in the granular layer (*p* = 0.240) nor in the molecular layer (*p* = 0.735).

### Cholinergic Innervation of the DG

**Figure 2:**
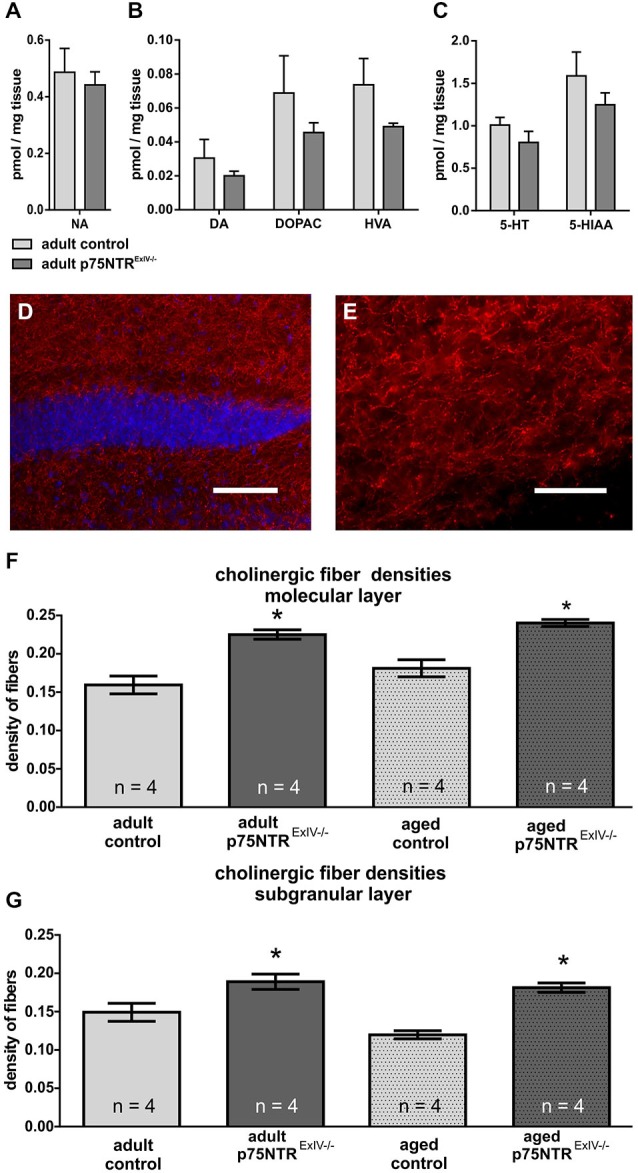
**p75NTR^ExIV−/−^** mice display an increased cholinergic innervation. Within the hippocampus, no major alteration were found in **(A)** noradrenaline (NA), **(B)** dopamine (DA) and its metabolites 3, 4-dihydroxyphenylacetic acid (DOPAC) and homovanillic acid (HVA) or **(C)** serotonin (5-HT) and its metabolite 5-hydroxyindoleacetic acid (5-HIAA). Using an antibody directed against ChAT, the cholinergic innervation within the DG can be visualized. ChAT-positive fibers are shown in red, whereas DAPI (in blue) was used to visualize cell nuclei [upper blade of the DG, around Bregma −2.18 mm, overview in **(D)**, higher magnification of the molecular layer in** (E)**]. Cholinergic fiber densities were increased in the molecular **(F)** as well as in the granular layer **(G)** of adult p75NTR^*ExIV−/−*^ mice. The differences in the altered cholinergic innervation persist in aged animals **(F,G)**. Scalebars: panel **(D)** = 100 μm; panel **(E)** = 50 μm.

We first analyzed the integrity of dopaminergic, noradrenergic or serotonergic systems within the hippocampus of p75NTR^*ExIV−/−*^ mice. Surprisingly, no major alterations concerning tissue concentrations and metabolism of the monoaminergic neurotransmitters such as NA (Figure 2A), DA (Figure 2B), and serotonin (Figure 2C) were found (for statistics, see Table 1).

**Table 1:** **Statistical analysis of the data obtained by the HPLC measurements**.

	control	P75NTR^ExIV−/−^	Difference	*t*	*P* value
NA	0.4867	0.4419	−0.04475	0.2997	*P* > 0.05
Dopamin	0.03048	0.02000	−0.01048	0.07015	*P* > 0.05
DOPAC	0.06881	0.04548	−0.02333	0.1562	*P* > 0.05
HVA	0.07361	0.04896	−0.02465	0.1650	*P* > 0.05
5-HIAA	1.588	1.248	−0.3401	2.278	*P* > 0.05
5-HT	1.009	0.8030	−0.2060	1.380	*P* > 0.05

*Bonferroni’s multiple comparisons test p75NTR^ExIV−/−^ vs. control (HPLC). Abbreviations: 5-HIAA = 5-hydroxyindoleacetic acid; 5-HT = serotonin; DA = dopamine; DOPAC = 3, 4-dihydroxyphenylacetic acid, HVA = homovanillic acid; NA = noradrenaline*.

We next examined whether and to what extent deletion of p75NTR^*ExIV*^ may affect the cholinergic system within the DG. In Figure 2 the data concerning altered cholinergic innervation in adult and aged p75NTR^*ExIV*^ are summarized. Our results indicate that cholinergic fiber densities in the molecular (*p* = 0.002) and subgranular (*p* = 0.041) layer of the DG in p75NTR^*ExIV*^ knockout mice were significantly increased by more than 32%, as compared with age-matched controls (Figure 2F). Thus, deletion of the short, in addition to the long receptor isoform, does not increase cholinergic hyperinnervation beyond the level seen in mice with a deletion of the long receptor isoform only (+30%; cf.; Dokter et al., 2015). The increase in cholinergic fiber density persisted in aged p75NTR^*ExIV−/−*^ mice (Figure 2F), in contrast to the p75NTR^*ExIII*^ knockout mice (Dokter et al., 2015), in the molecular)*p* = 0.003) as well as subgranular layer (*p* = 0.0003) of the DG.

### Cell Death within the Adult Dentate Gyrus

**Figure 3:**
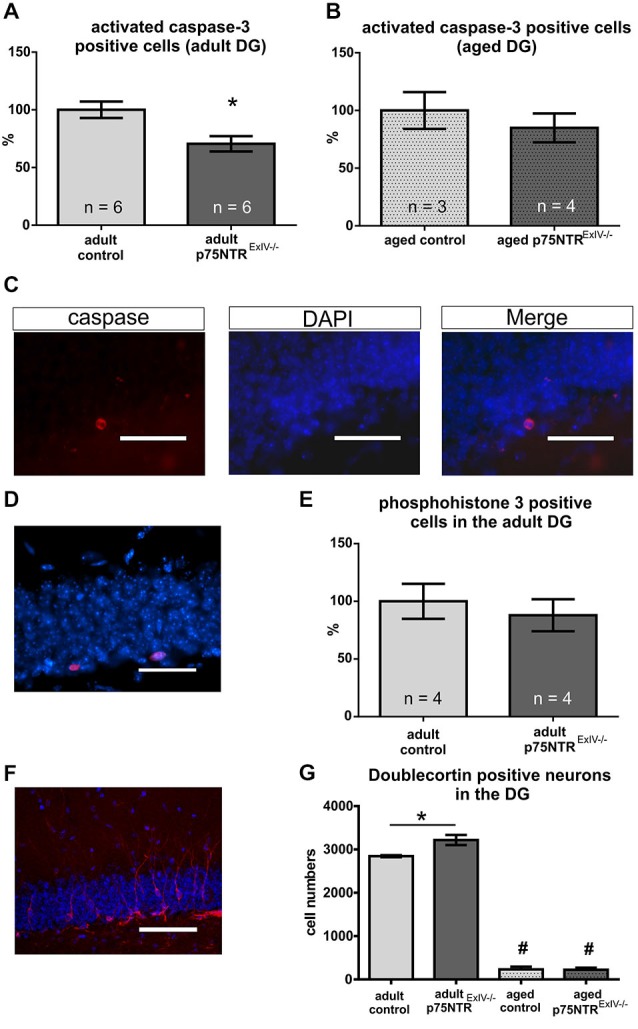
**Impact of a deletion of p75NTR on apoptosis, proliferation and differentiation**. Apoptotic cell death was monitored using caspase 3. Adult p75NTR^*ExIV−/−*^ mice display a strong reduction in the rate of apoptosis in the DG **(A)**. Aged p75NTR^*ExIV−/−*^ mice still display reduced rates of apoptosis; however, not significantly different from aged controls (**B**). In** (C)** an example of a caspase 3 immuno-positive cell within the DG is displayed. Cell proliferation in the adult DG was analyzed using phosphohistone H3 (PH3) immunohistochemistry [**D**; PH3 positive cells in red; DAPI (in blue was used to visualize cell nuclei)]. No significant alteration in the number of PH3-positive cells could be determined by comparing adult p75NTR^*ExIV−/−*^ and age-matched controls **(E)**. For analysis of the neuronal lineage doublecortin (DCX) was used as a specific marker (**F**; DCX positive neuron are shown in red; DAPI (in blue) was used to visualize cell nuclei). During aging, there is a dramatic reduction in the number of DCX-positive cells **(G)** as compared to adult animals of the same genotype (significant changes are indicated by #). Adult p75NTR^*ExIV−/−*^ mice have significant more DCX positive neuronal cells than age-matched controls (One-way ANOVA followed by a Tukey’s multiple comparisons test). Scalebars: panel **(C,D)**: 50 μm; panel **(E)**: 100 μm.

We have recently shown that the rates of cell death in the hippocampus of p75NTR^*ExIII*^ knockout mice are reduced as compared to controls (Dokter et al., 2015). Using activated caspase-3 as a marker for apoptosis; Figures 3A–C) we found that p75NTR^*ExIV*^ knockout mice display a slightly stronger reduction of apoptotic cell death in the DG as compared to age-matched controls (Figure 3A; *p* = 0.013). Aged p75NTR^*ExIV−/−*^ mice also showed reduced rates of apoptosis, but due to the great variance observed in older animals, the difference was not significant from age-matched controls (Figure 3B; *p* = 0.484).

### Adult Neurogenesis within the Dentate Gyrus

To further assess possible alterations in adult neurogenesis, we first studied the effects of a deletion of both, the truncated and long isoform of p75NTR on cell proliferation within the adult DG using phosphohistone H3 (PH3) as a marker (Figure 3D). In adult mice, the number of PH3 positive cells within the DG of p75NTR^*ExIV−/−*^ mice was not different from the number determined in control mice (*n* = 4 per group; *p* ≤ 0.58; Figure 3E). Likewise, aged p75NTR^*ExIV−/−*^ showed no alteration in the number of PH3 positive cells as compared to age-matched controls (*n* = 4 per group; *p* ≤ 0.12). Thus, neither deletion of Exon III (Dokter et al., 2015) nor of Exon IV of the p75NTR gene affects cell proliferation within the DG.

We next analyzed alterations within the early neuronal lineage by using doublecortin (DCX) as a marker for both mitotically active and postmitotic young neurons (Figure 3F). As shown in Figure 3G, adult p75NTR^*ExIV−/−*^ mice harbor significantly more DCX positive cells within the DG than age-matched p75NTR^*ExIV+/+*^ mice (*n* = 6; *p* = 0.01). This is in contrast to mice with a deletion of Exon III (Dokter et al., 2015).

In line with previous reports (Kuhn et al., 1996; Klempin and Kempermann, 2007) neurogenesis declines with age (Figure 3G). Similar to p75NTR^*ExIII−/−*^ mice (Dokter et al., 2015), aged p75NTR^*ExIV−/−*^ mice did not display significant alterations in the number of DCX-positive cells as compared with aged-matched controls (*n* = 3; Table 2). The overall increase in the population of DCX-positive cells in adult mice prompted us to analyze this effect in more detail.

**Table 2:** **Statistical data from the analysis of doublecortin (DCX) positive neurons within the DG**.

	Mean Diff.	95% CI of diff.	Significant?		
control vs. p75NTR^*ExIV−/−*^	−373.7	−669.6 to −77.84	Yes (*)		
control vs. aged control	2613	2250 to 2975	Yes (***)		
p75NTR^*ExIV−/−*^ vs. aged p75NTR^*ExIV−/−*^	2994	2632 to 3357	Yes (***)		
aged control vs. aged p75NTR^*ExIV−/−*^	8.000	−410.4 to 426.4	No		
**Test details**	**Mean 1**	**Mean 2**	**Mean diff**.	**q**	**DF**
control vs. p75NTR^*ExIV−/−*^	2845	3218	−373.7	5.192	14
control vs. aged control	2845	232.0	2613	29.64	14
p75NTR^*ExIV−/−*^ vs. aged p75NTR^*ExIV−/−*^	3218	224.0	2994	33.97	14
aged control vs. aged p75NTR^*ExIV−/−*^	232.0	224.0	8.000	0.0786	14

*Tukey’s multiple comparisons test p75NTR^ExIV−/−^ vs. control (Doublecortin)*.

**Figure 4:**
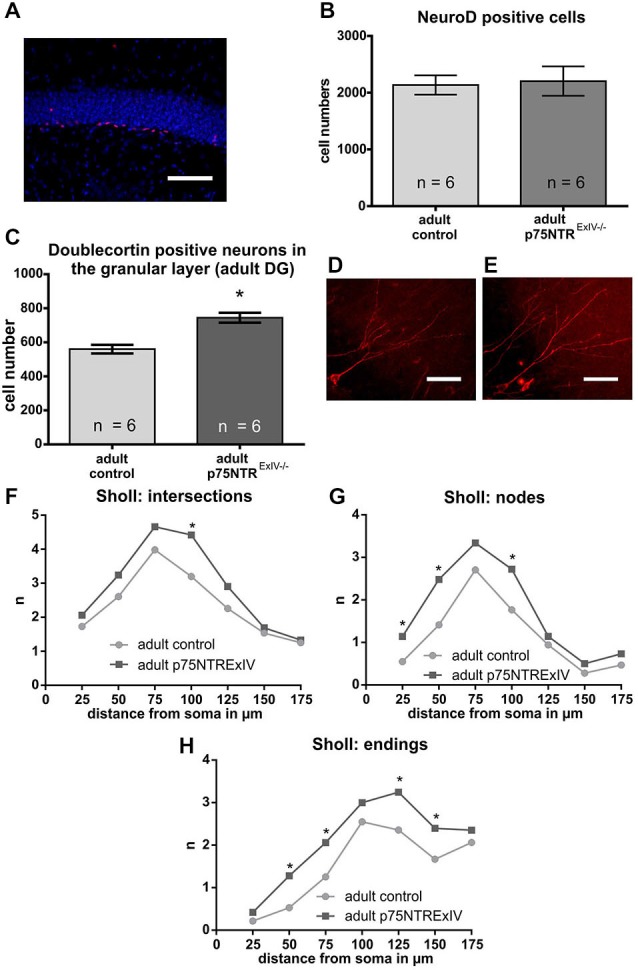
**Impact of a deletion of p75NTR on the neuronal lineage**. Mitotically active young neuronal cells were determined using NeuroD as a specific marker (**A**; the NeuroD positive cells are shown in red. DAPI-staining (in blue) was used to visualize cell nuclei). No significant differences in the number of NeuroD positive cells was noted **(B)**. The analysis of the postmitotical DCX-positive neurons revealed that adult p75NTR^*ExIV−/−*^ mice display significant more DCX neurons of that subpopulation than age-matched controls **(C)**. A comparison of images of late DCX-positive neurons in the adult DG that extents its dendrites towards the molecular layer of adult p75NTR^*ExIV+/+*^ mice **(D)** and p75NTR^*ExIV+/+*^ mice **(E)** hinted for a possible difference in their morphologies (NeuroLucida: deep projection of a z-stack). Sholl analysis revealed that the morphologies of those DCX-positive neurons were different [concerning intersections **(F)**, nodes **(G)** and endings **(H)**]. Scalebars: 100 μm.

First, the number of mitotically active young neurons was determined using NeuroD (von Bohlen und Halbach, 2007). As shown in Figure 4A, the number of NeuroD positive cells within the DG of p75NTR^*ExIV−/−*^ mice (2205 ± 259.8; *n* = 6) was not statistically different (*t*-test: *p* = 0.83) from the numbers determined in the respective controls (2136 ± 165; *n* = 6; Figure 4B).

Since the number of NeuroD positive cells remained unchanged, we next asked whether the observed phenotype regarding the increase in DCX positive cells might be attributable to an increase in the number of *postmitotic* young DCX-positive neurons. To estimate the number of those cells, only DCX positive cells were counted that are located within the granular layer of the DG and extend their DCX-positive dendrites towards the molecular layer of the DG. Our analysis revealed that p75NTR^*ExIV−/−*^ mice have significantly more postmitotic DCX positive young neurons than p75NTR^*ExIV+/+*^ mice (p75NTR^*ExIV−/−*^: 744.7 ± 29.14; *n* = 6; p75NTR^*ExIV+/+*^: 560.1 ± 25.17; *n* = 6; *p* = 0.0007; Figure 4C). Thus, we assume that deletion of both, the truncated and full-length isoform of p75NTR affects the differentiation of newly generated neurons. Since DCX-positive neurons of p75NTR^*ExIV+/+*^ mice (Figure 4D) seemed to have different morphologies as compared with DCX-positive neurons of p75NTR^*ExIV−/−*^ mice (Figure 4E), Sholl-analyses were conducted. Indeed, as shown in Figures 4F–H, DCX-positive neurons of the two genotypes displayed different morphologies, which was apparent in the number of dendritic intersections, nodes and endings. Thus, the morphologies of DCX positive neurons of p75NTR^*ExIV−/−*^ mice appear to be more complex than DCX positive neurons of control animals (see for statistics: Table 3).

**Table 3:** **Summary of the statistical differences (Sholl-analysis)**.

Distance from soma (μm)	Nodes	Intersection	Ending
25	0.0016	0.0760	0.0954
50	0.0013	0.0689	0.0012
75	0.0637	0.0613	0.0106
100	0.0052	0.0022	0.2494
125	0.4572	0.1856	0.0065
150	0.2396	0.7658	0.0295
175	0.5393	0.9084	0.5276
200	0.3019	0.4108	0.5586

*Sholl-analysis (*t*-test: *p*-values)*.

### Dendritic Spines of Granule Cells in the Dentate Gyrus

**Figure 5:**
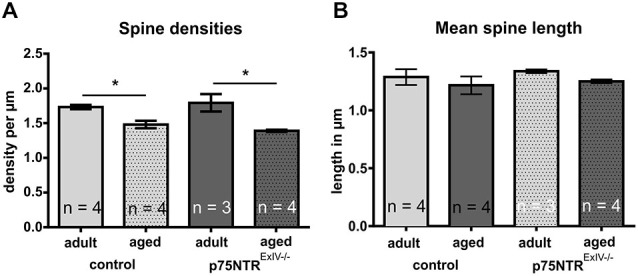
**Dendritic spines of mature granule cells in the DG**. **(A)** Adult or aged p75NTR^*ExIV−/−*^ mice did not differ from age-matched controls concerning the spine densities in the DG. Only an age-related decline in spine densities was noted in both, p75NTR^*ExIV−/−*^ mice and controls. **(B)** Neither age nor genotype induces a significant change in the mean spine length of granule cells of the DG.

Since p75NTR deficiency has been reported to affect dendritic spine densities of CA1 pyramidal neurons in organotypic cell cultures derived from p75NTR^*ExIII−/−*^ as well as p75NTR^*ExIV−/−*^ mice (Zagrebelsky et al., 2005), we next investigated whether p75NTR^*ExIV−/−*^ mice display alterations in dendritic spine densities of mature granule cells. Adult p75NTR^*ExIV−/−*^ mice, in contrast to adult p75NTR^*ExIII−/−*^ mice (Dokter et al., 2015), display similar spine densities as age-matched controls, whereas in aged p75NTR^*ExIV−/−*^ as well as p75NTR^*ExIII−/−*^ (Dokter et al., 2015) knockout animals no impact on spine densities within the DG was found. Furthermore, in both, p75NTR^*ExIV−/−*^ mice and age-matched controls, an age-related decline in spine densities was observed (Figure 5A). Thus, in controls, the spine densities declines significantly (*p* = 0.007) during aging from 17.33 to 14.81 spines per 10 μm and in p75NTR^*ExIV−/−*^ mice an age-related decline in spine densities from 17.93 to 13.71 spines per 10 μm (*p* = 0.011) was found. Concerning the mean length of dendritic spines, subtle alteration in mean spine length was noted by comparing p75NTR^*ExIV−/−*^ mice with age-matched controls; however, these effects were not significant (Figure 5B).

## Discussion

Previous studies have shown that deletion of the p75 neurotrophin receptor affects hippocampal morphology, adult neurogenesis and hippocampus-related behavior (Wright et al., 2004; Catts et al., 2008; Bernabeu and Longo, 2010; Colditz et al., 2010). However, most studies were done on p75NTR^*ExIII*^ deficient mice, which still express s-p75NTR (Nykjaer et al., 2005) and revealed contradictionary results. To overcome this obstacle, we used mice with a deletion of Exon IV resulting in a loss of both, the full-length and the short isoform of p75NTR (von Schack et al., 2001). Here, we show that p75NTR^*ExIV*^ deficient mice display morphological alterations in the adult DG, including an increase in thickness of the molecular and the granular layer of the DG.

Given that the DG is one of the two brain structures possessing a life-long regenerative capacity, we were interested in determining the morphological substrates that might account for the observed phenotype, since the cholinergic system, among others, may positively modulate adult hippocampal neurogenesis (Bruel-Jungerman et al., 2011). The hippocampus receives several inputs from the basal forebrain cholinergic system. It is known that deletion of p75NTR in mice leads to an increase in the number of cholinergic neurons in the septum. This has been demonstrated for p75NTR^*ExIII−/−*^ mice as well as for p75NTR^*ExIV−/−*^ mice, although the rise in cholinergic neuron numbers was found to be more pronounced in p75NTR^*ExIV−/−*^ mice (Naumann et al., 2002). We were able to show that the cholinergic innervation is also increased in the DG of p75NTR^*ExIV−/−*^ mice, which is likely to be a consequence of the increased numbers of cholinergic forebrain neurons. In contrast to p75NTR^*ExIII−/−*^ mice (Dokter et al., 2015), this increase in cholinergic innervation persisted throughout age in the p75NTR^*ExIV−/−*^ mice.

Acetylcholine is known to play an important role in learning and defects of the cholinergic systems are associated with aging and Alzheimer’s disease (AD; Muir, 1997). Age-related decline of memory function is accompanied by morphological alterations in the hippocampus (von Bohlen und Halbach and Unsicker, 2002) along with enhanced expression of p75NTR (Costantini et al., 2006). Brains affected by AD suffer from a severe decline of the cholinergic system (Terry and Davies, 1980) and p75NTR is thought to be involved in the pathogenesis of AD, including formation of tangles in the process of AD, mediating amyloid-β toxicity and stimulating amyloidogenesis (Hu et al., 2002; Chakravarthy et al., 2012). Interestingly, hippocampal p75NTR levels have been found to be increased in AD (Chakravarthy et al., 2012) and previous reports indicate that polymorphism in p75NTR are associated with a decreased risk for AD (Cozza et al., 2008; Cheng et al., 2012). Based on our current data, one could speculate that reduced expression of p75NTR in aged animals protects from age-related decline of the cholinergic system. It would be of great value to further determine whether p75NTR deficiency protects from the formation of tangles in AD pathogenesis.

Selective neurotoxic lesion of forebrain cholinergic input, e.g., with 192 IgG-saporin, was reported to reduce adult neurogenesis within the DG (Mohapel et al., 2005; Fréchette et al., 2009). Thus, we followed the idea that adult neurogenesis might be altered in p75NTR^*ExIV−/−*^ mice, thereby contributing to the morphological alterations of the DG. However, no effect was seen on the level of cell proliferation. Adult p75NTR knockout mice had increased numbers of newly formed DCX-positive neurons. In contrast to the cholinergic innervation, we were not able to detect a substantial increase in the numbers of DCX positive cells in aged p75NTR^*ExIV−/−*^ mice. Since adult p75NTR^*ExIV−/−*^ mice showed significant signs for elevated levels of neurogenesis, we next analyzed the population of DCX positive cells in more detail. Our results indicate that deletion of both forms of p75NTR increases the population of young postmitotic DCX-positive neurons. Moreover, p75NTR^*ExIV*^ deficient mice not only have elevated numbers of newborn neurons, but also more complex dendritic trees within that cell population, indicating altered neuronal differentiation.

P75NTR was shown to be expressed in the DG (Barrett et al., 2005) and recent fate-mapping experiments revealed its expression by progenitor cells located in the subgranular zone (SGZ) and by cells of the neuronal lineage (Bernabeu and Longo, 2010). Therefore, the effects on the population of DCX-positive cells might not be solely related to alterations of the cholinergic system, but also directly related to a cell-autonomous function of p75NTR in newborn cells. In line with this hypothesis, neurotrophins have been reported to induce apoptosis of hippocampal neurons via p75NTR signaling (Friedman, 2000; Troy et al., 2002). Additionally, p75NTR acts as a high-affinity receptor for proNGF, which has been described to have pro-apoptotic effects in the hippocampus (Guo et al., 2013). Death signaling is mediated via intracellular binding partners of the p75NTR, resulting in c-jun kinase activation and subsequent activation of p53, Bax-like proteins and caspases (reviewed in: Underwood and Coulson, 2008). Thus, one could expect that p75NTR deficient mice should display reduced rates of cell death. Nevertheless, in a study from 2008, increased rates of cell death were shown to be present in p75NTR^*ExIII*^ deficient mice (Catts et al., 2008). This prompted us to determine the rate of cell death in the DG of p75NTR^*ExIV−/−*^ mice by using activated caspase-3 as a specific marker. Using this approach, we were able to demonstrate that p75NTR^*ExIV−/−*^ mice hold reduced rates of apoptosis in the DG.

Both, reduced apoptosis and increased neurogenesis is likely to contribute to the observed thickening of the granule layer. Moreover, the increase of cholinergic fibers together with the enhancement of dendritic complexity might be partly the cause for the increase in molecular layer thickness. As mentioned earlier, increased dendritic complexity was shown to be present in hippocampal pyramidal neurons of organotypic cell cultures derived from p75NTR^*ExIV−/−*^ mice, but not in pyramidal neurons from p75NTR^*ExIII−/−*^ mice (Zagrebelsky et al., 2005), indicating that only deletion of both, the full-length and short isoform of p75NTR induces this phenotype at least in organotypic cell cultures. This study also indicated a marked increase in dendritic spine densities in pyramidal neurons in organotypic cell cultures derived from p75NTR^*ExIV−/−*^ mice (Zagrebelsky et al., 2005). We therefore speculated that spine densities of granule cells might also be affected in adult p75NTR^*ExIV*^ knockout mice. However, deletion of both, the full-length and the short isoform of p75NTR does not affect spine densities in granule cells in the knockout mice.

It would be interesting to further characterize the cell-specific contribution of p75NTR downstream targets such as Homologue of enhancer of split 1 and 5 (Hes1/5) and neurogenin 3, which might contribute to the phenotypes observed. Activation of p75NTR was shown to up-regulate the expression of Hes1/5 and increased expression of these genes is sufficient to decrease the number of dendrites (Salama-Cohen et al., 2005). Impairments in NGF/p75NTR activation on the other hand can lead to high levels of neurogenin 3, which was shown to stimulate dendritic outgrowth (Salama-Cohen et al., 2006). In addition, BDNF signaling, a well-established mediator of dendritic arborization seems to signal through cypin to regulate dendrite number (Kwon et al., 2011). Thus, it may be possible that in p75NTR^*ExIV−/−*^ mice, cypin promoted microtubule assembly (Tseng and Firestein, 2011) may be altered.

Using conditional p75NTR deficient mice, in which p75NTR was conditionally deleted in postmitotic choline-acetyl-transferase (ChAT) expressing cells, a lasting increase in the number of cholinergic neurons was observed (Boskovic et al., 2014), comparable to results shown for p75NTR^*ExIII*^ and p75NTR^*ExIV*^ deficient mice (Naumann et al., 2002). Contrary as to p75NTR^*ExIII*^ (Yeo et al., 1997; Greferath et al., 2012; Dokter et al., 2015) and p75NTR^*ExIV*^ mice, cholinergic innervation of the hippocampus is not altered in the ChAT-crep75^in/in^ mice (Boskovic et al., 2014). The ChAT-crep75^in/in^ mice, in contrast to p75^*ExIII*^ mutant mice (Catts et al., 2008; Dokter et al., 2015) display no alterations in the Morris water maze. Thus, it is possible that the changes in performance of the p75NTR^*ExIII−/−*^ mice are due to elimination of p75NTR from the developing and/or adult hippocampus (Boskovic et al., 2014) and subsequent effects are directly associated with p75NTR in the hippocampus, as e.g., in adult neurogenesis. In this context, it would be beneficial to analyze whether the observed morphological changes in the DG of the p75NTR^*ExIV*^ knockout mice translate into hippocampal dependent behavior. Most hippocampus related behavioral test require that the animals have to move or navigate properly (e.g., dark-light box, Morris water maze). However, due to hind limb ataxia p75NTR^*ExIV−/−*^ mice are not well-suited for those behavioral tests (von Schack et al., 2001).

In summary, our data show that deletion of both, the short and the full-length isoform of p75NTR causes altered DG morphology with an overall thickening of the involved cell layers.

Consequently, alterations in morphogenic substrates such as the quantity of cholinergic innervation as well as the amount of active neurogenesis and programmed cell death were evident. Thus, p75NTR is likely to play a role in regulating the cholinergic system and modulating adult neurogenesis by balancing neuronal differentiation and apoptotic cell death within the DG.

## Conflict of Interest Statement

The authors declare that the research was conducted in the absence of any commercial or financial relationships that could be construed as a potential conflict of interest.
